# HPV-Type Distribution and Reproducibility of Histological Diagnosis in Cervical Neoplasia in Poland

**DOI:** 10.1007/s12253-014-9877-4

**Published:** 2014-12-30

**Authors:** Andrzej Nowakowski, Sabrina Collas de Souza, Robert Jach, Dominique Rosillon, Alicja Książek, Katsiaryna Holl

**Affiliations:** 1 Department of Oncologic Gynecology and Gynecology, Medical University of Lublin, ul. Staszica 16, 20-081 Lublin, Poland; 2 Present Address: Department of Gynecology and Oncologic Gynecology, Military Institute of Medicine, ul. Szaserów 128, 04-141 Warsaw 44, Poland; 3 4Clinics, 8 rue de la Terrasse, F-75017 Paris, France; 4 Department of Obstetrics and Perinatology, Medical College of Jagiellonian University, Kraków University Hospital, ul. Kopernika 23, 31-501 Kraków, Poland; 5 GlaxoSmithKline Vaccines, Rue Fleming 20, 1300 Wavre, Belgium; 6 GlaxoSmithKline, ul. Rzymowskiego 53, 02-697 Warszawa, Poland

**Keywords:** Cervical cancer, CIN, Diagnosis reproducibility, HPV, Poland

## Abstract

This study was performed to assess attribution of high grade cervical intraepithelial neoplasia (HG-CIN) and invasive cervical cancer (ICC) to human papillomavirus (HPV) genotypes and secondarily to assess reproducibility of HG-CIN/ICC diagnosis obtained in Poland. Formaldehyde fixed, paraffin embedded blocks of HG-CIN/ICC from two distant institutions were sent to a central laboratory together with original histological diagnoses. Central/expert review of histopathological specimens was performed and agreement between local and central/expert diagnoses was calculated. HPV detection and genotyping in the samples was carried out with the use of SPF10-LiPA25 technology. Results were analyzed for 205 HG-CIN and 193 ICC cases with centrally confirmed diagnoses. Kappa coefficients and 95 % confidence intervals for HG-CIN and ICC diagnoses were: 0.13 (0.09;0.17) and 0.19 (0.11;0.26) respectively. Cohen’s kappa coefficients for lesions with representative number of samples ranged from 0.01 for cervical intraepithelial neoplasia grade 2 to 0.75 for adenocarcinoma. HPV DNA was detected in 96.1 and 91.2 % of the confirmed HG-CIN and ICC specimens respectively. HPV positive HG-CIN was most commonly attributed to HPV types: 16 (62.8), 33 (7.8), 31 (6.6), 52 (3.7), 45 (2.6) and 58 (2.6 %). HPV positive ICC was most commonly attributed to HPV types: 16 (72.1), 18 (10.8), 33 (5.7), 45 (3.4) and 31 (1.7 %). Reproducibility of histological diagnosis of HG-CIN/ICC obtained in Poland generally increases with the severity of lesion and is lowest for cervical intraepithelial neoplasia grade 2 and highest for adenocarcinoma. Over 80 % of ICC cases are vaccine-preventable in Poland.

## Introduction

Despite the long term opportunistic screening and initiation of active organized cytological screening in 2006 [1], cervical cancer (CC) age standardized mortality ratio of 5.8/100 000 in Poland in 2008 is estimated almost twice as high as the average 3.0/100 000 in the European Union [2]. Among possible causes are: low and stable coverage rates not exceeding 25 % of organized screening with unknown quality, undetermined coverage and quality of opportunistic screening, undetermined quality of triage of abnormal pap tests, the quality of histological diagnosis, treatment and follow-up of patients with cervical neoplasia [3].

Identification of high risk types of human papillomaviruses (HR HPVs) as a necessary etiological factor of CC has led to elaboration and implementation of effective vaccines against HPV in many countries around the world, however primary prevention of CC through HPV vaccination is still not available in the Polish reimbursed immunization program [4]. Information on HPV-type distribution in cervical neoplasia is crucial for pharmacoeconomic modeling of HPV vaccination impact on the epidemiology of cervical lesions. Such comprehensive and reliable data obtained on histological samples both of high grade cervical intraepithelial neoplasia (HG-CIN) and invasive cervical cancer (ICC) using standardized methodology have been unavailable for Poland yet. Available studies were performed on relatively small numbers of cases, cytological samples and/or performed with the use of older diagnostic methods [5–7].

The current study is a part of a large project which assessed the HPV-type distribution in HG-CIN encompassing CIN2, CIN2/3, CIN3 and adenocarcinoma in situ (AIS) and also in invasive cervical cancer (ICC) in selected European countries for which data were incomplete [8]. Our study has however focused only on Poland to provide up-to-date and detailed epidemiological information on the attribution of HG-CIN and ICC to certain HPV-genotypes specifically in the country.

One of the factors influencing the effectiveness of CC prevention programs and also crucial for clinical decision-making is the quality of histological diagnosis of cervical neoplasia. To our knowledge, data on this issue have not been published for Poland yet. For this reason we decided to use data collected in the international project [8] and performed a post-hoc analysis of reproducibility of HG-CIN and ICC histological diagnosis obtained in Poland. This type of analysis was not among the primary objectives of the international project and was not published in the primary paper [8].

In comparison to the primary manuscript [8], our work provides therefore detailed information on the attribution of HG-CIN/ICC to specific HPV genotypes and completely novel data on the reproducibility of pathological diagnosis in these lesions in Poland. The data collected in this study are necessary for accurate evaluation of cost-effectiveness of HPV vaccines and potentially to guide the selection of HPV tests for screening and triage of abnormal pap results in the screening program. Information on reproducibility of histological diagnosis of cervical neoplasia is important for implementation of quality reassurance measures in pathological work-up.

## Materials and Methods

### Study Design and Materials

Our study is based on the material collected in HERACLES and SCALE studies published recently [8] which were parallel, cross-sectional, multicenter studies of HPV-type distribution in 3103 women with HG-CIN and 3162 women with ICC, respectively, diagnosed in several European countries. In each participating site which maintained an archive of cervical excision tissue material, consecutive formalin-fixed paraffin-embedded excision specimens of HG-CIN and/or ICC diagnosed between 2001 and 2008 were collected. According to a standardized protocol of sample retrieval and collection, consecutive formalin-fixed paraffin-embedded tissue blocks from women diagnosed with HG-CIN and ICC between 2001 and 2008 were requested from the sites. Only most recent blocks containing the highest grade of CIN or the primary ICC obtained before chemo/radiotherapy were selected. Patient’s age at specimen collection, year of specimen collection and original diagnosis at site were recorded. To be eligible for the study, tissue blocks had to fulfill strict quality criteria [8]. Anonymized specimens were sent to the central laboratory (DDL Diagnostic Laboratory, Rijswijk, The Netherlands) for expert histopathological review and HPV genotyping. The study was approved by the central and local ethics committees and the patients (or their closest relatives if the patients were deceased) gave written informed consent for use of the tissue samples in the study.

Since this study focuses on HG-CIN/ICC diagnosed in Poland, the material used was collected at two distant sites in Poland: the First Department of Gynecology and Oncologic Gynecology of Medical University of Lublin and the Department of Gynecology and Oncology of the Jagiellonian University Medical College of Gynecology and Obstetrics in Krakow. The two sites participate in both organized and opportunistic cervical screening, perform triage of abnormal pap results, carry out treatment and follow-up of women with symptomatic and asymptomatic cervical neoplasia. Therefore tissue samples archived at these sites should be representative of the local population of women with cervical disease.

### Histopathological Review and Laboratory Analyses

To assure the presence of lesions in the material used for HPV genotyping, a “sandwich” method of material sectioning was applied. The first and the last of the four 4 μm thick sections was stained with haematoxilin and eosin (H&E) and reviewed by an experienced gynecological pathologist blinded to the original local diagnosis. Only if the presence and the type of the lesion was confirmed in both H&E sections, the two middle sections were used for DNA analysis. The highest grade of lesion present in the sample was taken into account in the analysis. The sectioning was performed with strict measures to avoid contamination [8]. Eligible sections were tested for the presence and type of HPV using SPF_10_-DEIA/LiPA_25_-polymerase chain reaction (PCR) system (SPF10-LiPA25; version 1, Labo Biomedical Products, Rijswijk, The Netherlands, based on licensed Innogenetics technology) [8]. Total DNA was isolated through proteinase K digestion and HPV DNA was amplified and detected with the use of SPF_10_-DEIA. In positive samples the HPV genotype was identified by reverse hybridization probe assay (SPF10-LiPA25), which detects 14 high-risk (HR) (16,18,31,33,35,39,45,51,52,56,58,59,66,68/73) and 11 low-risk (LR) HPV types (6,11,34,40,42,43,44,53,54,70,74). If the sample was HPV DNA negative, ten-fold DNA dilutions were retested. All HPV-negative and a random of HG-CIN and ICC samples were again reviewed by up to three expert pathologists blinded to HPV status. Cases were classified according to 2003 WHO classification [9]. If the diagnosis was discordant, a majority decision was taken into account, if all three expert pathologists disagreed, the case was rejected.

### Statistical Analysis

Statistical methods used in this study were different to those published in the primary manuscript [8] since no controlling for inter-country variability was required when calculating HPV-type distribution. Also additional analyses of reproducibility of HG-CIN/ICC diagnosis were performed since they were among the aims of this study.

Agreement levels for each diagnosis category of cervical lesions were calculated in percent. Simple and weighted kappa coefficients were calculated separately for HG-CIN and ICC cases because study procedures and analyses were done separately and cases were not transferred between the two groups [8]. The sample size for analysis of HPV type distribution in HG-CIN and ICC was predefined in such a way that the precision of the 95 % CI of the percentages would not exceed 5 % when the percentages were lower than 10 %. Attribution of lesions to specific HPV genotypes was calculated in HPV DNA positive cases. In each disease category, the minimal percentage attribution of the lesion to the HPV genotype was calculated as the frequency of single infections of that HPV genotype in this category of lesion. The maximal percentage of attribution was estimated by the frequency of single and multiple infections with the HPV genotype considered. In addition, we computed a percentage of proportional attribution of disease categories to HPV genotypes according to a previously described method [10, 11] where a case is proportionally attributed according to the frequency of the HPV type in the respective disease category. Statistical analyses were performed using SAS software (version 9.1).

## Results

**Fig. 1:**
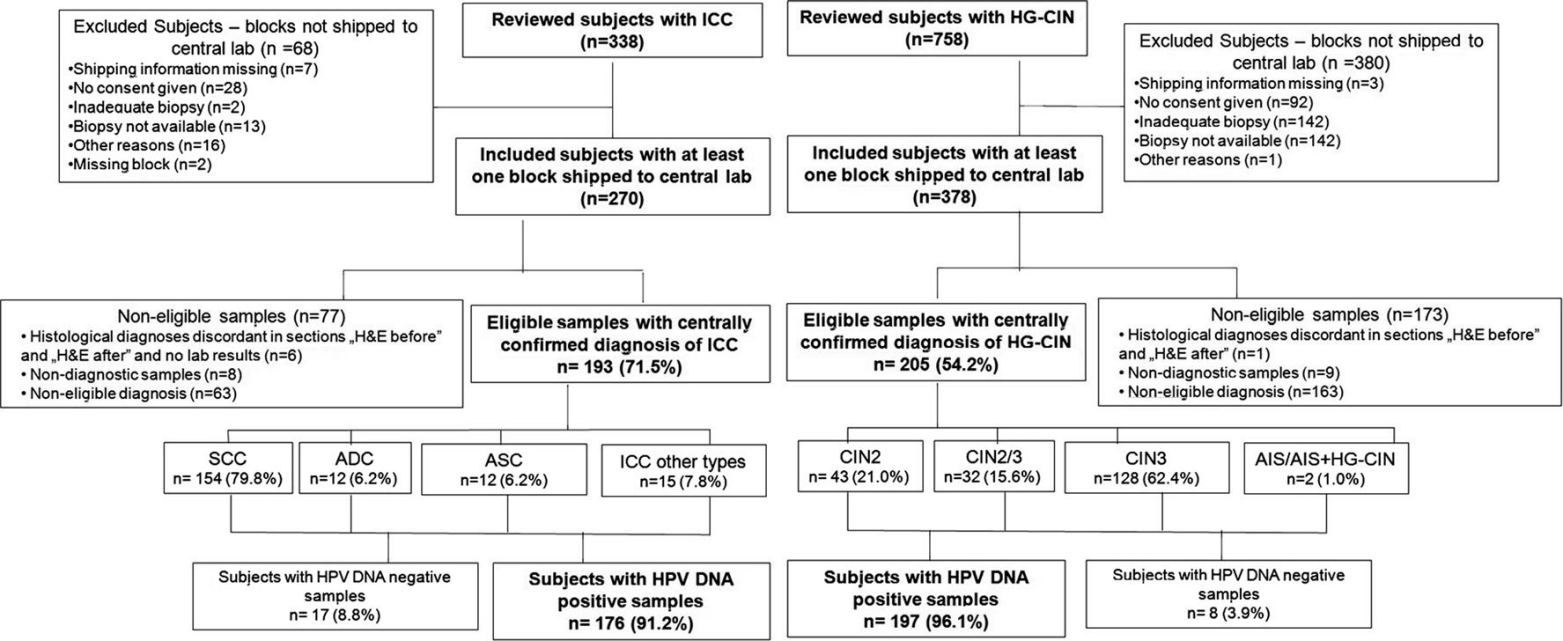
Flow chart of subjects with high grade cervical intraepithelial neoplasia (HG-CIN) and invasive cervical cancer (ICC). Abbreviations: *ICC* invasive cervical cancer, *HG-CIN* high grade cervical intraepithelial neoplasia, *SCC* squamous cell carcinoma, *ADC* adenocarcinoma of the cervix, *ASC* adenosquamous carcinoma of the cervix, *CIN2* cervical intraepithelial neoplasia grade 2, *CIN2/3* cervical intraepithelial neoplasia grade 2/3, *CIN3* cervical intraepithelial neoplasia grade 3, *AIS* adenocarcinoma in situ, *AIS + CIN3* adenocarcinoma in situ with cervical intraepithelial neoplasia grade 3 in one specimen. “ICC other types” include: 8 undifferentiated carcinomas, 6 microinvasive carcinomas and 1 neuroendocrine tumour. Percentages are computed out of the total number of subjects in the above category.

The flowcharts of study subjects with HG-CIN and ICC and their samples included in the analysis are presented on Fig. 1. Of 758 reviewed patients diagnosed with HG-CIN and 338 diagnosed with ICC between 2003 and 2008 at two study sites, 378 and 270 respectively were recruited into the study. Out of 378 HG-CIN specimens sent to the central laboratory, in 163 cases the central laboratory diagnosis was discordant with the local diagnosis of HG-CIN, 9 specimens were non-diagnostic and diagnosis was discordant between sections “before” and “after” the 2 middle sections planned for PCR in one case. Out of 270 ICC specimens, in 63 cases the central laboratory diagnosis was discordant with the local diagnosis of ICC, 8 specimens were non-diagnostic, 5 had no laboratory results, one had discordant diagnosis in sections “before” and “after” sections for PCR. As a result, paraffin blocks of 205 women with HG-CIN and 193 women with ICC fulfilled all study criteria required for inclusion into statistical analysis of molecular testing results. There were 43 (21.0 %) CIN2, 32 (15.6 %) CIN2/3, 128 (62.4 %) CIN3, 1 (0.5 %) AIS and 1 (0.5 %) AIS + CIN3 case in the HG-CIN group and 154 (79.8 %) squamous cell carcinomas (SCC), 12 (6.2 %) adenocarcinomas (ADC), 12 (6.2 %) adenosquamous carcinomas (ASC) and 15 (7.8 %) other histological types of tumours in the ICC group. Among 12 ADCs, there were: 10 (83.3 %) adenocarcinomas of the endocervix (ADC-CX), 1 (8.3 %) clear cell carcinoma (ADC-CC) and 1 (8.3 %) minimal deviation carcinoma (ADC-MIN). Among 15 cases with other histological types of cancer there were: 8 (53.3 %) cases of undifferentiated carcinoma of the cervix (UDC), 6 (40.0 %) microinvasive carcinomas (MIC) and 1 (6.7 %) neuroendocrine tumor (NEC). The median age in years (range) of women with HG-CIN was 37 (21–84) and specifically: 38 (25–84) for CIN2, 33 (24–77) for CIN2/3, 37 (21–73) for CIN3, 48 for AIS and 51 for AIS + HG-CIN. The median age (range) of women with ICC was 51 (25–86) and specifically: 51 (25–74) for SCC, 47 (40–69) for ADC, 56 (41–86) for ASC and 50 (31–80) for “other” diagnoses.

The agreement levels, simple and weighted kappa coefficients for local and central diagnoses of HG-CIN and ICC are presented in Tables 1 and 2. Kappa coefficient values (95 % confidence intervals) for individual diagnoses with representative numbers of cases were: 0.01 (−0.06;0.08) for CIN2, 0.36 (0.28;0.45) for CIN3, 0.20 (0.11;0.28) for SCC and 0.75 (0.56;0.93) for ADC.

**Table 1:** Agreement between local and central/expert diagnosis of high grade cervical intraepithelial neoplasia

	Central laboratory/Expert diagnosis									
Local diagnosis	Excluded^1^	<Eligible^2^	CIN2	CIN2/3	CIN3	AIS	AIS + CIN3	>Eligible^3^	Total	Agreement level (%)
CIN2	4	107	20	12	22	0	0	1	166	12.0
CIN2/3	0	3	0	1	1	0	0	0	5	20.0
CIN3	6	45	23	19	105	1	1	6	206	51.0
AIS	0	1	0	0	0	0	0	0	1	0

Data are numbers of subjects in each category; ^1^includes non diagnostic samples and samples with discordant diagnosis of sections “before” and “after” the ones sent for PCR; ^2^ includes: samples negative for cervical intraepithelial neoplasia, samples with cervical intraepithelial neoplasia grade 1, samples with glandular lesions less severe than adenocarcinoma in situ; *CIN2* cervical intraepithelial neoplasia grade 2, *CIN2/3* cervical intraepithelial neoplasia grade 2/3, *CIN3* cervical intraepithelial neoplasia grade 3, *AIS* adenocarcinoma in situ, *AIS + CIN3* adenocarcinoma in situ with cervical intraepithelial neoplasia grade 3 in one specimen; ^3^includes any invasive neoplasm. Simple Kappa coefficient = 0.13 (0.09;0.17), weighted Kappa coefficient = − 0.01 (−0.56;0.04). For computation of Kappa coefficients, cases with central diagnoses of AIS and AIS + CIN3 were combined. Central/expert diagnosed cases in: “Excluded”, “<Eligible”, “>Eligible” categories were combined since they had no equivalents in local diagnosis categories

**Table 2:** Agreement between local and central/expert diagnosis of invasive cervical cancer

Local diagnosis	Central laboratory/Expert diagnosis							
	Excluded^1^	Not eligible^2^	SCC	ADC	ASC	Other types^3^	Total	Agreement level (%)
SCC	14	58	152	0	8	14	246	61.8
ADC	0	3	0	11	2	1	17	64.7
ASC	0	1	2	1	2	0	6	33.3
UDC	0	1	0	0	0	0	1	0

Data are numbers of subjects in each category; ^1^includes non diagnostic samples, samples with missing diagnosis and with discordant diagnosis of sections “before” and “after” the ones sent for PCR; ^2^includes: samples with diagnoses of noninvasive lesions, endometrioid, not otherwise specified adenocarcinoma and other neoplasms (excluding undifferentiated, neuroendocrine and micro invasive carcinoma); *SCC* squamous cell carcinoma, *ADC* cervical adenocarcinoma, *ASC* adenosquamous carcinoma, ^3^includes undifferentiated, neuroendocrine and micro invasive carcinoma, *UDC* undifferentiated carcinoma. Simple Kappa coefficient = 0.19 (0.11;0.27), weighted Kappa coefficient = 0.38 (0.26;0.49). For computation of Kappa coefficients, cases with central diagnoses of “Excluded”, “Not eligible” and “Other types” were combined into one category

HPV DNA was detected in 197 (96.1 %) of the 205 eligible HG-CIN and in 176 (91.2 %) of the 193 eligible ICC specimens. HPV DNA was detected in 86.0 % of CIN2, 96.9 % of CIN2/3, 99.2 % of CIN3, 94.2 % of SCC, 83.3 % of ADC, 75.0 % of ASC and in 80.0 % of specimens with other histological types of ICC. The two cases of AIS and AIS + CIN3 were HPV-positive.

Among HG-CIN and ICC, there were 80.5 and 87.6 % samples with single HPV type respectively. In 12.7 % of HG-CIN and in 2.1 % of ICC multiple HPV types were detected. Undetermined HPV types were detected in 2.9 % of HG-CIN and 1.6 % of ICC. Minimal, proportional and maximal attributions of lesions to certain HPV genotypes are presented in Table 3.

**Table 3:** Attribution of HPV positive HG-CIN and HPV positive ICC to HPV genotypes

CIN2 *n* = 37	CIN2/3 *n* = 31	CIN3 *n* = 127	AIS *n* = 1	AIS + CIN3 *n* = 1	HG-CIN *n* = 197	HPV type	ICC *n* = 176	SCC *n* = 145	ADC *n* = 10	ASC *n* = 9	Other# *n* = 12
51.0 (45.9–51.4)	44.9 (35.5–45.2)	70.8 (59.8–71.7)	–	100.0 (100.0–100.0)	62.8 (53.3–63.5)	16	72.1 (69.9–72.2)	73.7 (71.0–73.8)	50.0 (50.0–50.0)	44.4 (44.4–44.4)	91.7 (91.7–91.7)
2.7 (2.7–2.7)	6.5 (6.5–6.5)	0.8 (0.8–0.8)	100.0 (100.0–100.0)	–	2.5 (2.5–2.5)	18	10.8 (10.8–10.8)	7.6 (7.6–7.6)	40.0 (40.0–40.0)	44.4 (44.4–44.4)	–
8.5 (8.1–10.8)	7.7 (3.2–9.7)	5.9 (4.7–8.7)	–	–	6.6 (5.1–9.1)	31	1.7 (1.7–1.7)	1.4 (1.4–1.4)	–	–	8.3 (8.3–8.3)
–	11.6 (9.7–12.9)	9.3 (8.7–12.6)	–	–	7.8 (7.1–10.2)	33	5.7 (5.7–6.3)	6.3 (6.2–6.9)	–	11.1 (11.1–11.1)	–
–	–	2.4 (2.4–2.4)	–	–	1.5 (1.5–1.5)	35	1.1 (1.1–1.1)	1.4 (1.4–1.4)	–	–	–
3.6 (2.7–5.4)	–	0.0 (0.0–0.8)	–	–	0.6 (0.5–1.5)	39	0.6 (0.6–0.6)	0.7 (0.7–0.7)	–	–	–
2.7 (2.7–2.7)	12.1 (9.7–12.9)	0.0 (0.0–0.8)	–	–	2.6 (2.0–3.0)	45	3.4 (3.4–3.4)	3.4 (3.4–3.4)	10.0 (10.0–10.0)	–	–
2.7 (2.7–2.7)	–	0.9 (0.8–3.1)	–	–	1.1 (1.0–2.5)	51	–	–	–	–	–
7.2 (5.4–8.1)	3.5 (3.2–6.5)	2.7 (2.4–6.3)	–	–	3.7 (3.0–6.6)	52	0.6 (0.6–1.1)	0.7 (0.7–1.4)	–	–	–
0.0 (0.0–2.7)	0.0 (0.0–3.2)	1.6 (1.6–2.4)	–	–	1.2 (1.0–2.5)	56	0.6 (0.6–0.6)	0.7 (0.7–0.7)	–	–	–
5.4 (5.4–5.4)	–	2.4 (2.4–3.1)	–	–	2.6 (2.5–3.0)	58	0.6 (0.6–1.1)	0.7 (0.7–1.4)	–	–	–
–	4.0 (3.2–6.5)	–	–	–	0.6 (0.5–1.0)	59	–	–	–	–	–
2.7 (2.7–2.7)	3.2 (3.2–3.2)	1.6 (1.6–2.4)	–	–	2.1 (2.0–2.5)	66	0.6 (0.6–0.6)	0.7 (0.7–0.7)	–	–	–
–	0.0 (0.0–3.2)	–	–	–	0.0 (0.0–0.5)	68/73	0.6 (0.6–1.1)	0.7 (0.7–1.4)	–	–	–
2.7 (2.7–2.7)	–	–	–	–	0.5 (0.5–0.5)	6*	–	–	–	–	–
–	–	–	–	–	–	11*	–	–	–	–	–
–	–	–	–	–	–	34*	–	–	–	–	–
–	–	–	–	–	–	40*	–	–	–	–	–
2.7 (2.7–2.7)	–	–	–	–	0.5 (0.5–0.5)	42*	–	–	–	–	–
–	–	–	–	–	–	43*	–	–	–	–	–
0.0 (0.0–2.7)	–	–	–	–	–	44*	–	–	–	–	–
2.7 (2.7–2.7)	0.0 (0.0–3.2)	–	–	–	0.5 (0.5–1.0)	53*	0.0 (0.0–0.6)	0.0 (0.0–0.7)	–	–	–
0.0 (0.0–2.7)	–	0.0 (0.0–0.8)	–	–	0.0 (0.0–1.0)	54*	–	–	–	–	–
–	–	–	–	–	–	70*	–	–	–	–	–
–	–	–	–	–	–	74*	–	–	–	–	–

Data are proportional attribution and minimal-maximal attribution ranges (in brackets) presented in %. Proportional attribution was computed according to frequency of the HPV type adjusted for multiple infections among the samples in the category of lesions. Minimal attribution was calculated as the percent of samples with single infection with the given type within the category of lesions. Maximal attribution was calculated as the percent of samples with certain HPV type as single and multiple infections within the category of lesions

*HG-CIN* high-grade cervical intraepithelial neoplasia, *ICC* invasive cervical cancer, *CIN2* cervical intraepithelial neoplasia grade 2, *CIN 2/3* cervical intraepithelial neoplasia grade 2/3, *CIN3* cervical intraepithelial neoplasia grade 3, *AIS* adenocarcinoma in situ, *AIS +* − adenocarcinoma in situ + any HG-CIN, *SCC* squamous cell carcinoma, *ADC* cervical adenocarcinoma, *ASC* adenosquamous carcinoma. # includes: neuroendocrine, undifferentiated and microinvasive squamous carcinoma;*-Low Risk HPV types

**Fig. 2:**
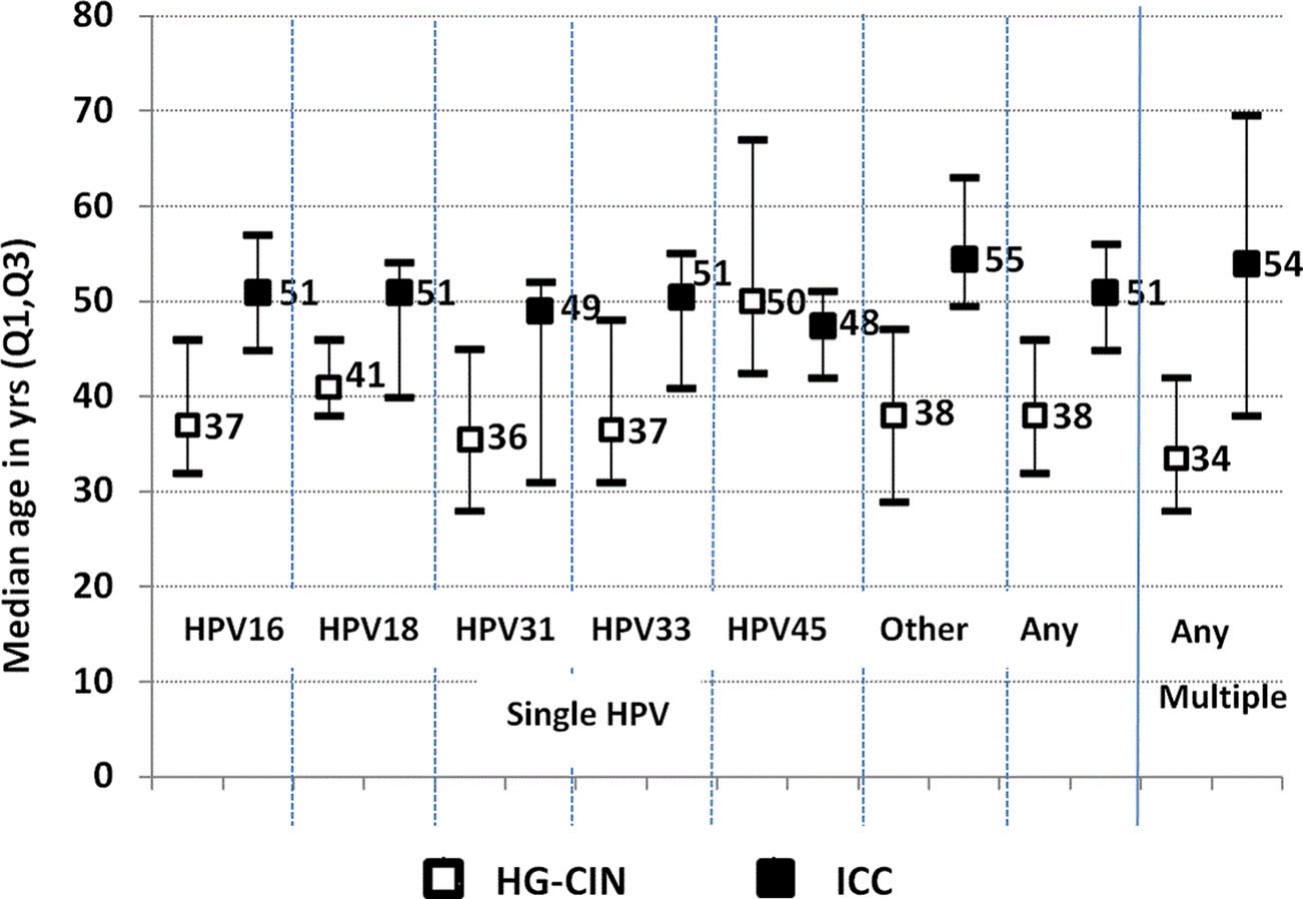
Median age at diagnosis of HG-CIN and ICC related to single type and multiple type HPV infections. Footnote: *HG-CIN* high grade cervical intraepithelial neoplasia, *ICC* invasive cervical cancer. “Other”: includes types other than: HPV16/18/31/33/45

Median ages (inter-quartile ranges) of patients at the time of histological specimen collection for women with HG-CIN and ICC positive for DNA of HPV: 16, 18, 31, 33, 45, other combined types and multiple types are presented at Fig. 2. Women with ICC were older than women with HG-CIN and the differences seem to vary with HPV genotype however p-values are not presented as this was a post-hoc analysis (Fig. 2).

## Discussion

To our knowledge this is the first comprehensive report on HPV prevalence and genotype distribution in almost 400 HG-CIN and ICC tissue specimens collected consecutively at two distant institutions in Poland and representative for local population of women. HPV DNA was detected in 96.1 % of HG-CIN and 91.2 % of ICC. HPV 16, 33, 31, 52 and 45 were found to be most commonly causally related to the development of HG-CIN and approximately 84 % of these lesions in Poland can be attributed to the five listed HPV genotypes. Low Risk HPVs (types 6, 42, 44, 53, 54) are responsible for less than 10 % of HG-CIN in Poland. In our series they were detected only in three CIN2 cases but absent in CIN2/3, CIN3 and ICC samples as single infections. The most frequent genotypes in ICC were: HPV 16, 18, 33, 45, 31 and over 80 % of ICC in Poland can be attributed to the first two genotypes. ICC cases were exclusively related to HR HPVs.

HPV DNA positivity rates of HG-CIN and ICC in Poland are very similar to rates observed for HG-CIN in our analysis of pooled European countries [8] and to rates for ICC published by de Sanjose et al. for the World [12]. The drop in HPV DNA positivity between tissue samples of HG-CIN and ICC was also noted in our European analysis [8]. It may be caused by HPV DNA integration to the host genome, degradation of HPV DNA – both resulting in the loss of sequences detected by PCR, and by other unclear factors.

HPV 16 and 33 were the most commonly found among polish HG-CIN samples both in our study and in a previous report by Kędzia et. al [5] but the order of the remaining genotypes is different and direct comparisons are impossible due to major methodological differences in design of both studies. The order of the four most common genotypes in the European [8] and in our series of HG-CIN specimens is the same (HPV 16, 33, 31, 52) however HPV 45 and HPV 18 were ranked fifth in Polish and the pooled European study respectively.

HPV 16 and 18 are universally the two most common genotypes among ICC in our Polish, European [8] and worldwide [12] series of samples. HPV 31, 33, 35, 45 and 52 are reported as 3rd to 5th most common genotypes with differing orders in our, another Polish [6], European [8] and the worldwide [12] studies.

Restrictive analysis incorporating only minimal attribution of ICC to HPV 16 and 18 obtained in our study reveals that theoretically 80.7 % of ICC cases are vaccine preventable in Poland. In the most optimistic scenario assuming maximal attribution of ICC to HPV 16 and 18 obtained in our study and a 50 % cross-protection of vaccines against HPV 31, 33 and 45, the rate of potentially vaccine preventable ICC cases in Poland would be close to 90 %. The data collected in this study are necessary for input into existing pharmacoeconomic models and analyses before decisions of health technology assessment regulatory agency in Poland on recommendations of reimbursement of HPV vaccination from public funds. Very high contribution of HPV 16 and 18 to the development of ICC in Poland is an opportunity for effective primary prevention of the great majority of invasive lesions. Unfortunately HPV vaccines are not yet reimbursed and immunization coverage is low.

In the light of our results indicating very frequent prevalence of HPV 16 and 18 among patients with ICC compared to HG-CIN in Poland, the newest recommendations on the use of HPV16/18 genotyping in the triage of women with atypical squamous cells of undetermined significance (ASC-US) or no intraepithelial lesions or malignancy (NILM) but a positive cocktail HPV test [13] are of great importance and may have profound clinical implications. The use of a test allowing for distinguishing between HPV16/18 and other types could substantially help in proper identification of women with normal/borderline cytology who require colposcopy as they are at highest risk of existing or developing cancer.

To our knowledge, this is the first literature report of the reproducibility of histological diagnosis of cervical neoplasia in Poland. The quality of CIN and ICC diagnosis is important for effective screening and treatment of cervical disease. Histological overdiagnosis of cervical lesions may result in overtreatment with subsequent higher rate of complications and adverse outcomes in the reproductive health [14]. On the other hand, underdiagnosis may result in undertreatment with profound negative outcomes, especially in cases of true invasive lesions. Our data confirm previous reports on high inter-observer variability in some categories of histological diagnosis of cervical neoplasia e.g. in CIN2 [15–17]. The level of agreement between local and central/expert diagnosis in our study seems to increase with the severity of cervical lesions. Also in discordant cases, local overdiagnosis was more common than underdiagnosis. Small numbers of rarer histological types of tumours do not allow for conclusions. The kappa coefficient was the lowest for CIN2 which is in agreement with previous reports [15, 16] of poor reproducibility of CIN2 diagnosis which might reflect that this type of lesion does not correspond to a well-defined phase of the pathogenic pathway of infection and transformation of the epithelial cells [15]. Dichotomous classification of CIN: CIN1 vs CIN2+ provides more reproducibility [15]. In our study local diagnosis of CIN2 was changed by the central review in great majority of samples which indicates that morphologic criteria of histological assessment are very subjective in cases of intermediate intraepithelial cervical pathology. We advocate the search for accurate biomarkers to facilitate dichotomous classification of cervical intraepithelial lesions into: 1) high probability of spontaneous regression, 2) medium to high probability of progression to cancer.

CIN2/3, according to WHO International Statistical Classification of the Diseases and Health Problems 10th edition [18], is not a separate entity however it was included in the original study protocol [8] and therefore included in our analysis. Among the few available cases of CIN2/3, local *vs* central diagnosis was fairly concordant.

Despite very low reproducibility of CIN2 and low-to-moderate reproducibility of CIN3 between local and central diagnosis in our study, invasive lesions were found by central/expert pathologists only in 1 (0.6 %) case of CIN2 and 6 (2.9 %) of CIN3 cases. 64.5 % of CIN2, 60 % of CIN2/3 and 42.2 % of CIN3 lesions were downgraded by central review which suggests a tendency to overdiagnosis and possible risk of overtreatment of a number of patients treated at our centers. However the most alarming finding is downgrading of almost a quarter of local site diagnosis of SCC. It is possible that extensive treatment protocols, overtreatment and adverse outcomes in some patients occurred. Blinding of the tissue blocks sent for study procedures to the central laboratory required by the central study protocol precludes identification of the over-treated patients. Strikingly, our results indicating possible overtreatment of CIN and ICC as a result of histological overdiagnosis are in contrast with epidemiological data of relatively high mortality and case-fatality rate of women with cervical cancer in Poland [19]. Low coverage and suboptimal quality of organized screening and triage, subsequent diagnosis of many women at advanced/symptomatic stages of ICC and suboptimal treatment are among the possible causes of the unfavorable epidemiological situation in Poland.

Our results indirectly indicate that time of progression of HG-CIN to ICC may vary according to HPV genotype causing the lesion (Fig. 2). However we interpret these findings with caution being aware of cross-sectional design of the study and limited number of samples infected with some HPV types.

The strengths of the part of our study devoted to HPV detection and genotyping include the use of a standardized study protocol, consecutive collection of histological samples representative for local populations at two distant gynecologic oncology centers participating in all steps of national active and opportunistic cervical screening and providing comprehensive diagnosis and treatment of CIN and ICC. Central expert histopathological review of histological material from 2003 to 2008 and the use of a highly sensitive HPV genotyping method provide an up-to-date picture of the attribution of HG-CIN and ICC to specific HPV genotypes in Poland.

The apparent drawback of our analysis, which should be addressed in future studies, is the lack of analysis of reproducibility of our local histological diagnosis of non-neoplastic epithelium (normal, inflammatory, atrophic, reactive etc.) in cases suspected of cervical neoplasia (e.g. based on cytology/colposcopy/HPV testing results) and estimation of falls-negativity rate of our local histology. This information could have profound clinical implications however assessment of the quality of pathological work-up was not among the end-points of the main protocol [8] and we were only able to assess reproducibility of local diagnosis of cervical neoplasia, as a post-hoc analysis.

Nevertheless, further personnel training, improvement of quality control measures and possibly a wider use of biomarkers such as p16 (INK4a) and MIB-1 [20–22] could help to increase the quality of histological diagnosis of cervical neoplasia in Poland. HPV 16, 33 and 31 are the most common etiological factors of HG-CIN in Poland. However in ICC, HPV 16 and 18 are the biggest players and are together responsible for the development of over 80 % of lesions in Poland. This information obtained in our study is important for future vaccination programs and HPV-based screening protocols.
